# The effect of staining and bleaching on the color of two different types of composite restoration

**DOI:** 10.4317/jced.58837

**Published:** 2021-12-01

**Authors:** Shatha Kh. Hussain, Sarah W. Al-Abbasi, Majed-Mohamed Refaat, Abdullah M. Hussain

**Affiliations:** 1 University of Basrah, College of Dentistry, Restorative and Esthetic Dentistry Department, Assistant Lecturer, Basrah, Iraq; 2 University of Basrah, College of Dentistry, Preventive dentistry Department, Lecturer, Basrah, Iraq; 3 University of Basrah, College of Dentistry, Prosthetic dentistry Department, Lecturer, Basrah, Iraq; 4 University of Basrah, College of Dentistry, Restorative and Esthetic Dentistry Department, Assistant Lecturer, Basrah, Iraq

## Abstract

**Background:**

Long term success of composite restorations depends greatly on their color stability and esthetic appearance. This study aimed to assess the effects of commonly consumed beverage and bleaching on the color of composite restoration.

**Material and Methods:**

Two resin composite were used Filtek™ Z350 XT (3M/ESPE) and Briliant EverGlow (Coltene). Fifteen discs were made from each resin composite then baseline color measurements were made. The specimens divided into 3 groups (n=5) according to the storage solution. The storage solutions that used in this study are distilled water, tea and coffee. The specimens were stored in the solutions for 3h/day for 40 days and then second color measurements were done. Specimens were bleached by using of 40% H2O2 Opalescence boost. Then the specimens color were measured for the third time. For measuring color change CIE L* a* b* system was used. The results were statistically analyzed using Two-way analysis of variance (ANOVA) and Bonferroni tests. Level of significance was set at *p* < 0.05.

**Results:**

In all groups there were a significant color change (∆E>3.3). The discs that were made from Filtek™ Z350 XT (3M/ESPE) and immersed in coffee show the highest color change whereas the discs that were made from Briliant EverGlow and stored in distilled water show the least color change. Significant improvements in the color of specimens were demonstrated after bleaching, however bleaching couldn’t restore the composite color to clinically acceptable level (∆E<3.3).

**Conclusions:**

Both tested materials were susceptible to staining in the three-staining solution; Filtek™ Z350 XT showed more staining susceptibility than Briliant EverGlow. In office bleaching reverse some of the effect of staining from the surface of the composite but it couldn’t restore the composite color to its original color before staining.

** Key words:**Staining, color change, resin composite, bleaching.

## Introduction

Since the first time that the composite restoration had released to dental market, composite restoration had used broadly as a restoration for both anterior and posterior teeth. In spite of continuous improvement in the properties of composite materials, there are still some problems associated with the use of composite. In addition to polymerization shrinkage and secondary caries, color change is one of the major problems associated with composite usage (1,2).

Discoloration represents the major cause for replacing the composite restoration especially in the anterior teeth. Color stability of the restoration is very important to keep the esthetic appearance of the teeth and to satisfy the patient’s demand (3).

In order to serve as long –term esthetic restorative material composite should maintain its color and polish over a long period. Many factors can result in discoloration of composite restoration. Color change of composite restoration may be due to intrinsic or extrinsic factors (4). intrinsic discoloration can be defined as discoloration in the component of the composite. Extrinsic discoloration is result from deep or superficial absorption of staining substance after long-term exposure to that substance (5).

Patients are usually asked the dentists about how long the esthetic restoration should last and if their dietary habit influence on the longevity of these restorations (6). It has been found that the hygiene, smoking and eating habits result in discoloration of composite (7). For that reason the maintenance of esthetic restorations depend on the patient’s lifestyle and dietary habits (6).

Over long-term of exposure to different types of drinks and food materials, composite restorations tend to stain and change their colors (8). Nowadays there is an increase in the consumption of soft drinks and coffee (7). Coffee, tea and carbonated drinks are considered as the most commonly used drinks that can cause discoloration of resin composite (8).

The invention of newer composite materials is the result of continuous researches in the field of material science, however these composite materials differ in their structure (9). The introduction of the nanotechnology in the field of conservative dentistry led to the development of nanofilled and nanohybrid composite. Nanohybird composite resin contains a mixture of fillers that have micro and nano sized and their diameters are 0.3–1 μm and 0.02–0.05 μm, respectively. On the other hand, nanofilled composite resin has nano sized filler particles which are both single (nanomer) and cluster. The size of nano particles varies from 5–75nm (10). Nono-sized fillers allow better finishes of surface and a smooth texture which provides a natural appearance for the restoration (11).

Using of the visual techniques for the assessment of the color of restoration is subjective. In order to eliminate the subjectivity of visual techniques many of electronic devices had been introduced including colorimeter and spectrophotometer. These electronic devices evaluate the color of restorations using (International Commission on Illumination (CIE L*a*b* color system) (12).

The Aim of this study was to evaluate the effect of two popular drinks (coffee and tea) on the color stability of two commercially available nanohybrid resin composites. In this study we evaluate the effect of bleaching agent on the color of stained composite and if bleaching can restore the color of the composite near to the baseline color.

## Material and Methods

-Specimens preparation

For this *in vitro* study two commercially available types of composite were used with A2 shade and thirty discs were prepared. Fifteen discs were made from Filtek™ Z350 XT (3M/ESPE), and the other fifteen discs were made from Briliant EverGlow(Coltene/ Whaledent AG Altstatten, Switzerland). In order to make the discs a plastic mold was used and the mold had a thickness of 1.5 mm and a diameter of 8 mm (11). The plastic mold was placed over Mylar strip on a glass slide. The mold filled with the tested composites then another Mylar strip and glass slide were placed over the mold (13,14). In order to obtain flat and smooth surface a weight of 500Kg was placed over the glass slide. The polymerization of the specimens was done with wide spectrum curing LED unit from Fanta for 40 seconds. When the discs removed from the mold they were finished and polished using Enhance finishing points and polishing cups (Enhance System, Dentsply Caulk, U.S.A) (15). To ensure complete polymerization all of the specimens were immersed in distilled water at 37◦C for 24 hours (15,16). A total of fifteen discs from each composite were divided into three subgroups randomly (n=5).3

-Baseline color measurements

For color measurement a spectrophotometer (Vita Easyshade Advance, Vita Zahnfabrik, Bad Säckingen, Germany) was used. Before color measurement the specimens were placed over white background in order to prevent any potential color absorption effect during color measurement (17). For each disc three color measurement were made and the mean of the three readings was calculated and used in data analysis (18). The color measurements were done before and after staining and after bleaching. The specimens color was expressed according to the CIE l*a*b* system (14). The axis L* represents the lightness coordinate whereas the axes a* and b* are chromaticity coordinates. The lightness value ranges from zero (black) to 100 (white), while a* is the red-green axis and b*is the yellow-blue axis. When a* values is positive the color shift to red and negative values mean it shift to green. Positive b* values is represent the yellow range of the color and negative values represent the blue range of the color (19).

-Staining procedure

A total of fifteen discs from each composite were divided into three subgroups randomly (n=5) according to the storage solution. The storage solutions that used in this study are distilled water, tea and coffee (14). Tea solution is prepared by adding 1 teabag 2.0 g (Lipton,Yellow Label, Unilever United Arab Emirate) for 10 minutes into 150ml of boiled distilled water (14). In order to prepare coffee solution 3.6mg of coffee(Nescafe Classic, Nestle, Switzerland) was dissolved in 300ml of boiled distilled water, after stirring for 10 minutes the coffee was filtered with filtering paper. The specimens were placed in the solutions for 3hours after which they were cleaned with distilled water, and immersed in distilled water. This was done for 40 days, and the solutions were changed daily (2,8). The manufacturer of coffee claimed that the time that required to drink a cup of coffee is 15 minutes and coffee consumer drink from 2 to 3 cups per day. So the storage for 3h/day for 40 days is equivalent to 5 months of coffee consumption (2). At the end of 40 days the specimens were washed with distilled water and air dried, after which, another color measurement with spectrophotometer were done as described earlier.

-Bleaching procedure

For bleaching 40% H2O2 Opalescence boost (Ultradent Products, Inc., South Jordan, Utah, USA) is used and it was placed over the polished surface of the specimens for 3 times and the duration of each application is 15 minutes, so the total time of bleaching is 45 minutes. Between the applications the specimens were cleaned with distilled water and dried (13). When bleaching procedure was end the specimens were washed with distilled water for 1 minute and air dried before the third color measurement was taken (9).

-Color difference measurements

The color differences (∆E) between the three measurements were calculated by using the Hunter’s equation: (20).

ΔE = [(ΔL)2+(Δa)2+(Δb)2]1/2

ΔE represent the overall color difference, ΔL* is the difference in the lightness,

Δa* is the difference in axis a* of chroma and Δb* is the difference in axis of b* of chroma (3).

(∆E1) is the color difference from the baseline after staining, (∆E2) is the color difference between the color after bleaching and the color after staining, and (∆E3) is the color difference between the color after bleaching and the baseline.

ΔE<1 cannot detected by human eye and ΔE greater than 1 and less than 3.3 is considered noticeable but it is accepTable clinically while ΔE>3.3 is considered unaccepTable clinically (12,13).

-Statistical analysis

Data analysis used “Statistical package for social science (SPSS-21)” (Chicago, In Press)

Two-way ANOVA was used to test the effect of the brand, staining solution and bleaching on the color change (∆E1, ∆E2 and ∆E3) Also Two-way ANOVA was used to evaluate the possibility of interaction between the two factors. Bonferroni correction was used as post-hoc test for pair-wise comparisons of values amongst different groups when the ANOVA tests were significant. The level of significance was set at *p* < 0.05.

## Results

-After Staining (ΔE1)

Mean and standard deviations for color change results are shown in Table 1. After staining all of the staining solutions(coffee, tea, distilled water) for both of the resin composite restorative material brands used in this study show color change which is considered unaccepTable clinically (∆E>3.3). The discs that were made from Filtek™ Z350 XT (3M/ESPE) and immersed in coffee show the highest color change whereas the discs that were made from Briliant EverGlow and immersed in distilled water show the least color change in comparison to other groups (Table 1).

**Table 1:**
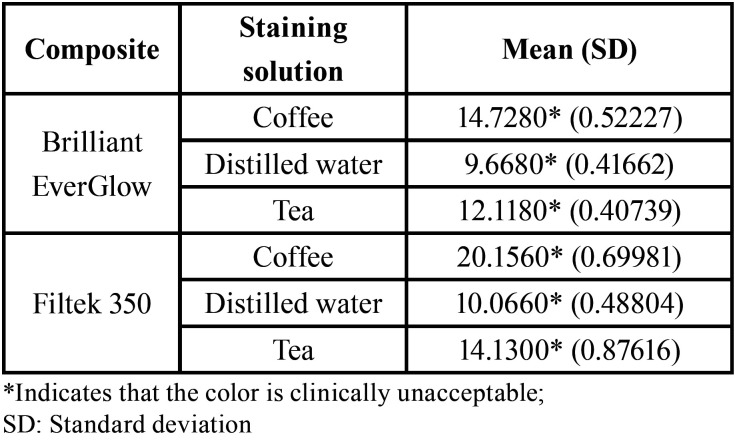
Mean (SD) of color change (ΔE1).

2-way ANOVA) showed that there were significant effects of the discoloration of the restorative material brand and storage solution, as well as their interactions (*p* < 0.001) on ΔE1 (Table 2).

**Table 2:**
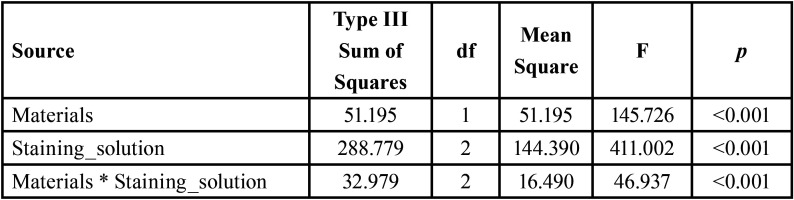
Two-way analysis of variance for resin composites and Staining solutions.

-After Bleaching (ΔE2)

The means and standard deviations of the color change of both resin composites in each immersion solution after bleaching are shown in (Table 3).

**Table 3:**
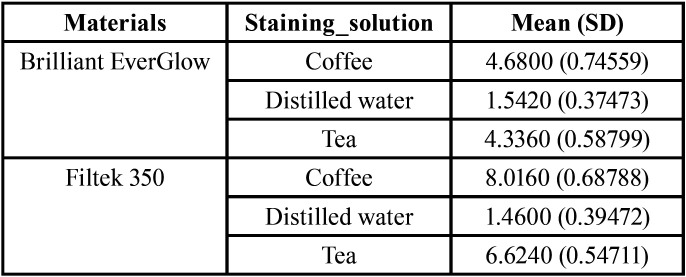
Mean (SD) of color change (ΔE2).

After bleaching for both of the resin composite restorative material brands the specimens stored in coffee and tea demonstrated color changes greater than 3.3 and specimens that were stored in distilled water showed color change less than 3.3.

After bleaching significant improvements in the color of specimens were demonstrated but when we compared the color after bleaching with baseline color we found that all groups showed (ΔE3) values above 3.3 (Table 4). The recovery effect of the bleaching agent couldn’t return the color of the composite to original color before staining.

**Table 4:**
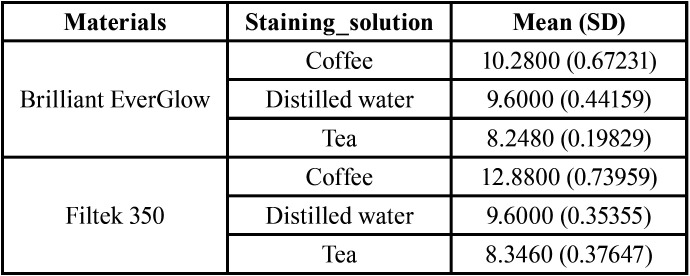
Mean (SD) of color change (ΔE3).

## Discussion

In this study we evaluate the effect of commonly used drinks on the color of two different commercially available resin composites. Also the effect of bleaching with 40% of hydrogen peroxide on the color of stained composites and its ability to restore the color to the baseline was evaluated.

Perception of color is a psychological issue and it greatly depends on the observer’s skill and may be perceived differently on different occasions. In order to overcome the errors associated with visual assessment of the color many of color evaluating devices has been used (21). In dentistry color evaluation is done by using Colorimeters and Spectrophotometers. These devices can measure the slight discoloration more precisely than the naked eye (22). Color measurement is usually done by using the CIELAB color system (23). CIEL*a*b system is very popular and it provides a useful, standardized technique for analysis of ΔE* values accurately (24). In this study we use the spectrophotometer and the CIe L*a*b* coordinates system which is in accordance with previous studies (11,12,16). The CIe L*a*b* system was chosen to determine the color difference (∆E) because it can determine small color changes precisely and have many advantages including sensitivity, repeatability and objectivity (6).

The discoloration of the composite can be caused by either extrinsic stain or intrinsic stain. Extrinsic stain can be divided into surface and subsurface stain. Surface stain is caused by food accumulation, beverages and plaque, while the subsurface stain is resulted from diffusing of surface stain into superficial resin layers and form chemical bonds with composite. Intrinsic stain is caused by reactions that occur within the composite component, this reactions is considered as physico-chemical interaction (25). Staining process of dental composite can affected by many factors such as the type of resin matrix, filler size, degree of polymerization, degree of smoothness, water sorption, type and exposure time to staining solution. Also, environmental factors such as PH and the temperature can affect staining process (25,26).

In this study, we use coffee and tea as staining solution since they are very popular drinks. To stimulate susceptibility of restorations to staining the period of staining was kept at 3 h per day because it had been found that the average person needs approximating 60–180 min per day for drinking and eating (9).

In this study both composite resins showed color changes (>3.3) when stored in coffee, tea and distilled water. The coffee solution reported the greater color change when compared with the two other staining solutions (tea and distilled water). Coffee resulted in greater discoloration may be due to both absorption and adsorption of the yellow stains which have low-polarity. This low polarity yellow stain has the ability to penetrate into deeper layers of resin matrix (27,28). Tea has high polarity yellow stains which only adsorb and precipitate on the surface without penetration into the resin matrix (14). The finding of our study is in agreement with several studies (2,14,29) except for the part that related to the immersion in distilled water. In our study we found that immersion in distilled water demonstrated unaccepTable color change (>3.3) and this disagree with Farah and Elwi (14) who found that the immersion in distilled water resulted in imperceptible color changes (ΔE ≤ 1). This discrepancy could be due to differences in the duration of immersion.

In this study the two types of composite didn’t show the same color stability. The ability of resin composite to discolor depends on hydrophilicity of the matrix resin and the ability of composite to absorb water (3,29). Both the resin content of the resin composite and the type of the bond between the filler and the resin can have an effect on the amount of water sorption of the composite (29). Furthermore, the discoloration of the composite could be due to presence of high amount of unreacted monomers. This unreacted monomers indicates low degree of conversion, and results in higher solubility and decreased color stability (30).

Patient who is looking for bleaching treatment could have a restored tooth with different type of esthetic restorative materials including resin composite; therefore it is necessary to know what is the effect of the bleaching treatment on the properties of restorative materials including color stability. The effect of bleaching agent on the color of restorative material is of clinical significance because the change in the color can be perceived by the patient (31).

The bleaching procedure used in this study is a simulation of in-office bleaching by using of high hydrogen peroxide (40%) concentration which is a chemically activated system. In this study the use of 40% hydrogen peroxide on the stained composite resulted in clinically noticeable color change (>3.3) for specimens that immersed in coffee and tea, but the specimens that immersed in distilled water the color change was between greater than 1 and less than 3.3. When we compare the color after the bleaching with the baseline color ∆E was greater than 3.3 in all groups. This can be explained by that the bleaching agent is only removed the extrinsic stains that result from the immersion in the staining solution by weakening the bond between the stain and the composite which result in elimination of the stain (9). The result of this study is in agreement with Nahedh and Awlyia who found that the use of high concentration of hydrogen peroxide wasn’t able to return the color of the composite to clinically accepTable ∆E values. They attributed this to a short and acute attack of strong oxidizing agent which could have less effect than a longer treatment with a lower concentration of hydrogen peroxide (32).

In spite of the bleaching agents can remove the superficial stains from the composite but they can’t bleach the composite the same as they bleach tooth structure. Therefore, after the application of the bleaching agent, the composite restoration may not always have the same color of adjacent bleached tooth structure (33).

## Conclusions

It is concluded that all of the test groups demonstrated a significant color change after their storage in different staining solutions and the discoloration was greater than clinically acceptable value (ΔE > 3.3). The greatest color change was caused by coffee. After bleaching although there was a significant improvement in the color of all understudy specimens, but the color change was above 3.3, which means that in office bleaching may reverse some of the effect of staining from the surface of the composite but it couldn’t restore the composite color to its original color before staining.
